# Barriers to obtaining reliable results from evaluations of teaching quality in undergraduate medical education

**DOI:** 10.1186/s12909-020-02227-w

**Published:** 2020-09-29

**Authors:** Zemiao Zhang, Qi Wu, Xinping Zhang, Juyang Xiong, Lan Zhang, Hong Le

**Affiliations:** 1 School of Medicine and Health Management, Tongji Medical College, Huazhong University of Science and Technology, Wuhan, Hubei Province China; 2 Tongji Institute of Medical Education Research, Huazhong University of Science and Technology, Wuhan, Hubei Province China

**Keywords:** Medical education, Undergraduate, Evaluation of teaching quality, Issues unrelated to teaching, Interviews

## Abstract

**Background:**

Medical education is characterized by numerous features that are different from other higher education programmes, and evaluations of teaching quality are an integral part of medical education. Although scholars have made extensive efforts to enhance the quality of teaching, various issues unrelated to teaching that interfere with the accuracy of evaluation results remain. The purpose of this study is to identify the barriers that prevent objective and reliable results from being obtained during the evaluation process.

**Methods:**

This study used mixed methods (3 data sources) to collect opinions from different stakeholders. Based on purposive sampling, 16 experts familiar with teaching management and 12 s- and third-year students were invited to participate in interviews and discussions, respectively. Additionally, based on systematic random sampling, 74 teachers were invited to complete a questionnaire survey. All qualitative data were imported into NVivo software and analysed using thematic analysis in chronological order and based on grounded theory. Statistical analyses of the questionnaire results were conducted using SPSS software.

**Results:**

Sixty-nine valid questionnaires (93.24%) were recovered. A total of 29 open codes were extracted, and 14 axial codes were summarized and divided into four selective codes: evaluation preparation, the index system, the operation process, and the consequences of evaluation. The main barriers to obtaining reliable evaluation results included inadequate attention, unreasonable weighting, poor teaching facilities, an index without pertinence and appropriate descriptions, bad time-points, incomplete information on the system, lagged feedback, and disappointing result application. Almost all participants suggested lowering the weight of students as subjects, with a weight of 50–60% being appropriate. Students showed dissatisfaction with evaluation software, and the participants disagreed over the definition of good teaching and the management of student attendance.

**Conclusions:**

This study reveals the difficulties and problems in current evaluations of teaching in medical education. Collecting data from multiple stakeholders helps in better understanding the evaluation process. Educators need to be aware of various issues that may affect the final results when designing the evaluation system and interpreting the results. More research on solutions to these problems and the development of a reasonable evaluation system is warranted.

## Background

In recent years, major national reforms of postgraduate medical education have taken place in numerous countries, including reforms with regard to the requirements of teaching and assessment strategies [1]. China is no exception. On the one hand, as an interdisciplinary subject, medical education is related to the implementation of the “Healthy China” strategy; on the other hand, it is associated with the construction of “educational power”. To cope with the demands for scientific and technological revolution, in 2018, the Ministry of Education proposed constructing a “new medical science”. The “new medical science” concept stresses the importance of the quality of teaching and aims to create high-quality professional and “gold courses”. As the foundation of standardizing medical educational management, the *Standards for Basic Medical Education in China (2016 version)* emphasize the importance of evaluations of teaching quality, which involve a process of systemically gathering information to judge the effectiveness and adequacy of educational programmes to identify gaps and facilitate improvements. It is well known that assessment promotes improvement, and in medical education, evaluation is a fact of life [2]. Although many medical colleges have established a system for evaluating teaching quality, the reliability and validity of the final results are questioned [3–5]. What barriers prevent objective and accurate results from being obtained?

Faculty evaluation is generally considered a formal measure undertaken by the academic authorities in a college to assess the academic performance of faculty members, including all activities related to teaching, research, administration and service [6]. In this respect, the evaluation of teaching quality is only one part. Numerous studies have proposed many issues that may be related to the implementation of evaluations, such as people’s attitudes towards and perceptions of evaluations, evaluation methods and tools, evaluator characteristics, and the wording of evaluation items [7]. However, the impact of each issue is still controversial, which may be due to differences in the research environment and objects. For example, some researchers support student evaluations of teaching (SETs), while others examine the presence of bias and question the effectiveness of this approach [8, 9]. William Burton et al. suggest that both quantitative and qualitative data obtained from online evaluations are better than data collected from paper forms [10]. In contrast, some studies hold that the differences in scores between online evaluations and paper evaluations are not statistically significant. There are also several studies regarding the impacts of the participation rate on the evaluation results that draw contradictory conclusions [11].

However, such research is rarely conducted in the context of medical education. Medical education is different from other higher education programmes in many ways, including the course structure, instructional design, method selection and teacher arrangement, which will affect course evaluations [12–14]. For example, it is quite common for medical students to have a different lecturer for almost every session during the pre-clinical years. When evaluating such a curriculum, it is important not only to evaluate individual teachers but also to recognize greater issues such as the overarching organization of the curriculum, whether the order of topics is logical and whether there is redundancy in the content. Different choices of indicators may lead to different final teacher evaluation results [12, 15]. Taking a German medical school as an example, Sarah Schiekirka et al. explore the promoting and impeding conditions of the evaluation process based on student perceptions. In their view, students’ inadequate understanding of the teaching system may lead to deviation of the results. The proposed consequences to be drawn from evaluation results are mainly directed at individual teachers rather than institutions or teaching modules [16]. Brandl et al. insist that in medical education, discussion in a structured environment yields more useful feedback and better satisfaction compared to an online survey [17].

Moreover, previous studies have paid great attention only to students’ perceptions of evaluations of teaching, resulting in insufficient attention to peer teachers and administrative staff as evaluation subjects. Debroy et al. attempt to investigate teachers’ views on SETs rather than the whole evaluation system [18]. From the methodological perspective, most research consists of quantitative studies and focuses on a single variable, ignoring qualitative research methods. In a systematic review of issues that influence student ratings of medical course evaluations, only 2 qualitative studies were included [19]. Qualitative methods play an important role in determining causes and solving complex problems [20].

The literature review shows that in addition to the teacher’s own teaching level, there are many issues unrelated to teaching that affect the final evaluation results. This paper tries to identify the existing barriers that prevent objective and authentic results from being obtained during the process of evaluating teaching quality in the context of medical education in China. Different stakeholders’ views on teaching evaluation are collected using mixed methods and are analysed in detail. We hope that this research can help in better understanding the teaching evaluation process and serve as a reference for deepening the reform of medical education and improving the quality of teaching.

## Methods

### Study design

With a 112-year history, our medical college is one of the birthplaces of modern Chinese medical education. In recent years, our college has attached great importance to the reform of medical education and has strived to improve the quality of medical education. Our research team consists of professional teachers with concurrent administrative positions, including one male and two females. Considering the complexity of the problem, we decided to use mixed methods integrating both qualitative and quantitative elements to collect views from different stakeholders [21, 22]. The qualitative element of the study involved semi-structured interviews and focus group discussions among experts and students, respectively. Moreover, the quantitative element involved the use of a questionnaire survey among teachers. The survey instruments were developed by our professors in the research team based on the literature review and the actual situation of our college [12, 23]. Currently, there is no authoritative investigation tool for this topic. Conducting the literature review, we found that some interview guides included the purpose of evaluation, the definition of good teaching, the evaluation indicators, and the consequences of evaluation [16]. Some investigation forms were constructed based on the necessity of evaluation, people’s satisfaction with tools, the appropriateness of the time of evaluation, and the publication of the results [24]. At present, our college’s teaching evaluation programme focuses on the selection of the evaluation subjects and weight distribution as well as the application and feedback of the evaluation results. Therefore, based on a consideration of all the issues noted above, the research topic consisted of the definition of good teaching, people’s attitudes towards evaluations of teaching, the selection of the evaluation subjects and weight distribution, the evaluation indicators, the equipment used in the evaluation process, the time of the evaluations, and the application and feedback of the results. The questions might vary according to the survey object or survey form [25, 26]. For example, when the research objects were students, their experience with teaching evaluation software was examined. In fact, peer teachers usually complete a paper evaluation form instead of an online evaluation. Therefore, it does not make sense to ask teachers about their experience with evaluation software. We also invited every participant to make other comments on evaluations of teaching to prevent previously unexpected information from being missed.

Experts and students were invited to participate based on purposive sampling with the help of the Dean of Students Office via a cohort-wide e-mail, and everyone agreed to cooperate with our investigation and provided written informed consent. All interviews and discussions were hosted by the corresponding author and facilitated by the first two authors, who were trained in qualitative research and served as an observer and a note taker. During the introduction to each session, the interviewers shared only their roles and the purpose of this study with the participants to ensure the consistency and coverage of topics. A questionnaire survey was also conducted to collect the views of teachers via e-mail with the help of the Dean of Students Office. For this survey, we adopted systematic random sampling to select full-time professional teachers without any administrative positions from different schools. The study was approved by the university’s medical education research ethics committee. All field studies were conducted in our college from May 2019 to August 2019, and all researchers had no competing interests with the participants.

### Semi-structured interviews

Semi-structured interviews were conducted with 16 teaching management experts from different schools in their workplace at a convenient time; among them, 2 were deans of faculty and departments, 5 were heads of departments, 1 was head of the teaching office, and 6 were from the teaching supervisor group. Thirteen of these 16 experts had senior titles (professors and associate professors), accounting for 81.25% of the sample. Regarding the number of years teaching, 12 experts (75.00%) had been teaching for over 20 years, while 13 experts (81.25%) thought that they were familiar with the content. Prior to the formal interview, a pre-investigation was performed with two experts. Using feedback from the pilots, we finalized the interview guide. During the formal interview, the interviewer prompted the respondents to further elaborate if the responses were brief or unclear. Each interview lasted approximately 60 min until data saturation was reached. At the end of the interviews, the experts were also invited to list any missing items that might be important issues influencing the accuracy of evaluations of teaching. All experts were familiar with the school’s teaching regulations and had their personal views on existing problems. The interview guide is presented below (Table 1).

**Table 1:** Semi-structured interviews guide

Item	Question
1	How do you define the high-quality teaching?
	In process of teaching supervision, what aspects of the teacher do you pay more attention to? (only to teaching supervisor)
2	What do you think is the subject of teaching evaluation? How should the weights be distributed?
3	What are the guarantee conditions for teaching evaluation?
4	What do you think of the current evaluation index system?
5	How do you achieve a balance between teaching and research?
6	When do you think is the appropriate time for teaching evaluation?
7	Do you think the division of teaching evaluation results should be based on absolute value or percentage?
8	What do you think about the feedback of teaching evaluation?
9	On the application of evaluation results, which way do you support?
10	What difficulties have you encountered in teaching evaluation management?
11	Other issues or key points.

### Questionnaire survey

In addition to interviews, a questionnaire survey was conducted to collect the views of teachers via e-mail. We adopted systematic random sampling to select 74 teachers, including lecturers, associate professors and professors. The contents of the questionnaire covered the evaluation subjects, the use of the evaluation results, the time of evaluations of teaching, influencing factors (such as the number of lecturers), feedback of the results and other issues, with 13 items in total (Additional file 1). A draft of the questionnaire was pilot-tested with four teachers. Cronbach’s alpha was calculated to estimate the reliability of the instrument and was found to be 0.78. The survey itself was completely anonymous.

### Focus group discussions

Two focus group discussions involving 12 s- and third-year students (including 5 males and 7 females) were conducted. The invited students had participated in the mid-term teaching forum and had a certain understanding of evaluations of teaching. In this study, the participating students were informed of the topics and were presented an outline of the discussion in advance to ensure active participation, the independence of views and the depth of topic mining during the formal discussion. Compared with the interviews, the focus group discussions, conducted in a quiet classroom, lasted longer (approximately 90 min) until data saturation was reached. The students were also invited to list any missing items that might be important for the evaluation of teaching quality. The discussion guide is presented below (Table 2).

**Table 2:** Focus group discussion guide

Item	Question
1	How do you define the high-quality teaching?
2	What do you think is the subject of teaching evaluation? How should the weights be distributed?
3	Do you take the evaluation seriously every time?
4	What do you think of the current evaluation index system?
5	What do you think of the evaluation operation software?
6	When do you think is the appropriate time for teaching evaluation?
7	Do you get feedback on the results of the teaching evaluation?
8	In order to ensure the smooth progress of teaching evaluation, what kind of work did your school do?
9	On the application of evaluation results, which way do you support?
10	What difficulties have you encountered in the process of teaching evaluation?
11	Other issues or key points.

### Data analysis

The semi-structured interviews and focus group discussions were voice-recorded, transcribed verbatim and double checked by 2 main authors to guarantee no omissions. First, the experts and students were coded E and S for expert and student, respectively. Then, all qualitative data were imported into NVivo software and carefully analysed using thematic analysis in chronological order and based on grounded theory. Notably, grounded theory is a research method for constructing theory in qualitative research, compensating up for the overly stylized research process in empirical research. There are three main phases involved in the use of grounded theory for research: open coding, axial coding, and selective coding [27]. With an open attitude, interview data are analysed piece by piece. Once a conceptual similarity or relevance in meaning is found, it is used to gather more abstract concepts. Axial coding explores and establishes various relationships between concepts and categories through the coding paradigms of conditions, strategies, and results. Selective coding further deletes and integrates the previous research results, formulates story lines, and constructs a theoretical framework [27–29]. For example, when “emphasis on teaching quality” was mentioned extensively by the participants, it was selected as one open code, thus positioning it as a central category of the indicators. “Emphasis on teaching quality” was subsequently recorded as “attitude towards evaluations” (axial coding) when similar categories emerged from the data, such as “participation in teaching evaluations”. Considering that “attitude towards evaluations” and other axial codes were usually determined at the early stage of evaluating teaching, they were selectively coded for evaluation preparation. Using the preceding steps, the concepts, categories, and core categories were summarized, and the theoretical model was constructed. The professors were mainly responsible for the coding process. Expert review was used as a reliability strategy. One of professors performed the initial analysis of all transcripts, and then two other professors independently reviewed and coded the transcripts. Discrepancies in the interpretation of materials were resolved through constant comparisons and iterative discussions among the members of the research team until agreement was reached.

Regarding the questionnaire results, statistical analyses were conducted using SPSS 19.0 software, and numerical data were described in terms of their composition ratio, rate, mean, and variance.

## Results

Regarding the questionnaire survey, 69 valid questionnaires (93.24%) were eventually recovered. Among the participants, there were 35 men and 34 women. The numbers of professors, associate professors, and lecturers were 23, 25 and 21, respectively. Analysing the qualitative and quantitative data, we extracted 29 open codes that affected evaluations of teaching quality and summarized 14 axial codes, which were divided into four selective codes: evaluation preparation, the index system, the operation process, and the consequences of evaluation (Table 3).

**Table 3:** Coding analysis of barriers to obtaining reliable evaluation results

Conceptual refinement	Open coding	Axial coding	Selective coding
Pay attention to, negativity perfunctory, careless, etc.	Emphasis on teaching quality	Attitude towards evaluation	Evaluation preparation
Mandatory, reminder, course selection time limit, related to test results, etc.	Participation in teaching evaluation		
Research orientation, focus, energy, academic frontiers, etc.	Balance between research and teaching	Idea of evaluation	
Reform, bondage, indoctrination, etc.	Encouragement of innovative teaching methods/models		
Thinking logic, innovation, utilitarian, acceptance, etc.	Respect the major role played by students		
Experienced, objective, reference value, human relationship, etc.	Professionalism of the subject	Evaluation subject and weight	
Coverage, comprehensive, continuous, no time, etc.	Participation of the subject		
Responsible person, joint responsibility, hiring teacher, mobility, etc.	Number of instructors for the course	Targets for evaluation	
Overall effect, quality, teaching team, basic score, etc.	Convenience of teaching evaluation		
Smart classroom, projector, teaching software, etc.	Fluency in the use of teaching equipment	Teaching quality guarantee system	
Arrivals, fingerprints, attendance, etc.	Student attendance management		
Electives courses, required courses, theoretical courses, humanities, etc.	Type of the curriculum	Specific indicators	Index system
Standards, understanding, materialization, etc.	Indicator connotation		
Excessive, burdensome, simple, etc.	Indicator structure		
Concerns, points, special, etc.	Importance of the Indicator	Indicator weight	
Distinction, median, mean, maximum and minimum, fairness, etc.	Analysis mode	Calculation method	
Group teaching, false results, assignments, discussions, etc.	Course arrangement	Teaching performance	Operation process
Skilled, vivid, attractive, thinking, etc.	Capacity and effectiveness of teaching		
Truancy, discipline, lateness, etc.	Classroom management		
Evaluation in the classroom, remember, timeliness, immediate, QR code, link, etc.	Timeliness of evaluation	Evaluation time	
Mid-term, end-of-term, biased, not over, exams, etc.	Comprehensiveness of evaluation		
Updates, inconsistent conditions, incomplete information, no correspondence, etc.	Integrity of teacher information	Computer operating system	
Sensitive, flashback, mobile, experience, operation, etc.	Fluency in system usage		
Written, verbal, guided, merit, communication, after class, etc.	Teaching improvements	Feedback loop	Consequences of evaluation
Class management, deficiencies, tutor selection, Q & A, etc.	Emotion of teachers		
Material, motivation, assessment, reward and punishment, promotion, selection, etc.	Teacher motivation	Basis of rewards and punishment	
Excellence, improvement, stress, collective lesson preparation, etc.	Teacher growth		
Ask, improve, feel, self-evaluate, etc.	Initiative to improve	Follow up the correction and improvement	
Targeting, monitoring, focus, quality, etc.	Effectiveness of improvement measures		

### Evaluation preparation

#### Inadequate attention to teaching quality

*E1 “At present, our institution places more emphasis on scientific research. Teachers put a lot of energy into scientific research work and are less innovative in their teaching”.*

*E2 “Since I started teaching in 1993, many students have adopted a perfunctory attitude towards evaluations. They just think teaching evaluations have little to do with them”.*

*S10 “The more common situation is the one-time evaluation at the end of the semester, which is generally favourable for convenience”.*

Many interviewees complained that the university paid too much attention to scientific research output and ignored teaching quality management. Teachers lacked the motivation to engage in teaching innovation and to improve the quality of their teaching, and they were tired of coping with teaching evaluations conducted by peers. Undoubtedly, negative attitudes towards a course affect student ratings. Most students could not consciously and seriously evaluate the quality of teaching due to insufficient publicity and mobilization work. Our institution did not take strict compulsory measures; instead, it only provided links for “whether to check final grades” to “whether to evaluate teaching”. As a result, most students evaluated teaching at the end of the semester, and they generally gave good reviews for the sake of convenience. Most students did not even carefully read the indices in teaching evaluations, affecting the accuracy of the results.

#### The weight of students as subjects is too high

*E5 “The weight of 80% for students as subjects is so high, and 50% is more appropriate. The scoring given by students has a certain authenticity, but there are also unreasonable points. Handwriting mistakes and malicious scoring do exist. In terms of teaching methods, classroom management and other aspects, evaluations by peer teachers and supervisors are more professional”.*

Our institution takes students as the main subjects, currently assigning them a weight of 80%, and almost everyone agrees on the diversity of subjects. However, a number of respondents stated that the weight attribution of the subjects was unreasonable. The experts believed that there were individual differences in the judgement of teaching styles by college students, and students’ ability to judge the content of teaching was generally lacking. Based on the statistical results of the questionnaire survey, 98.39% of the teachers supported the diversity of subjects. Typically, the top three most recognized subjects were students (65, 94.20%), peer teachers (61, 88.41%), and supervisors (57, 82.61%), with weights of 42.72 ± 18.08%, 22.83 ± 12.14%, and 21.60 ± 9.00%, respectively. Additionally, some supported teachers themselves (39, 56.52%), teaching administrators (25, 36.23%) and department heads (11, 15.94%) as subjects (Table 4).

**Table 4:** Teacher’s opinions about the evaluation subjects and the weights

subject	n(*N* = 69)	Top 3 weights^a^ (weight%, Proportion)			Mean ± SD	Min.	Max.
		No.1	No.2	No.3			
Students	65	50(26.3%)	30(22.8%)	20(15.8%)	42.72 ± 18.08	10	90
Teacher themselves	39	20(19.3%)	10(17.5%)	30(7.0%)	23.47 ± 17.88	5	100
Peer teachers	61	20(35.1%)	30(14.0%)	10(12.3%)	22.83 ± 12.14	3	50
Supervisors	57	20(40.4%)	30(15.8%)	10(10.5%)	21.60 ± 9.00	5	40
Leaders	11	10(7.0%)	5(7.0%)	15/20(1.8%)	9.50 ± 4.97	5	20
Administrators	25	10(19.3%)	5(8.8%)	20(7.0%)	12.14 ± 8.15	5	40
Others	3	–			30.00 ± 14.14	20	40

Notes^a^: The weights are filled in by respondents, and the sum of weights is 100%

#### Poor teaching facilities

*E6“The teaching facilities are too poor. Computers and headsets frequently fail. The software used in class should be debugged one week in advance. The projection problem has not yet been solved, which will affect students' evaluations of teachers”.*

At present, the staff in our college lack awareness of equipment management, leading to insufficient maintenance of the teaching facilities. Only when the teaching equipment is used smoothly can the work of teaching be carried out normally. Any problem with equipment will not only limit the innovation of teaching performance but also cause unnecessary troubles for the normal organization of teaching.

### The index system

#### The index design is not targeted

*E16“The type of course determines the design of the index system. The indices for elective/compulsory courses and theoretical/experimental courses should be different”.*

*E9“Different subjects have different understandings of teaching, so different indices should be employed”.*

*E14“Student attendance should not be included in the index system. It should be managed by the Academic Affairs Office”.*

According to our investigation, the management of student attendance was controversial. Some experts believed that a high student attendance rate should be the responsibility of the instructor and should be included in the evaluation index system. Some experts held the view that it is up to students themselves and that they should be responsible. Other experts suggested that the school’s Academic Affairs Office should manage attendance in a uniform manner. The definition of “good” teaching also varied considerably among different individuals. Students fancied teachers with a cheerful character, preferred interactive teaching, and enjoyed a free and easy classroom atmosphere. Peer teachers tended to evaluate teaching from the perspectives of teaching methods and knowledge transmission. From the perspective of the whole college, supervisors paid attention to both teachers’ teaching and students’ learning. Teachers identified gaps between the expected and actual effects and motivated themselves through self-evaluation. However, under the current situation, the same index system is utilized for different subjects, resulting in unsuitable results.

#### The index statements are too empty

*E14“The index is not very operable and should be specified”.*

*S1“It is difficult to determine whether the teaching methods are good enough”.*

Another barrier to quality evaluation was that some indices were too general and ambiguous, preventing the evaluator from making a judgement. Students might not be able to understand the terminology of education, such as “situated learning”, “cognitive load”, and “competence”; thus, they relied more on their feelings to evaluate teaching quality. Students preferred scaled questions over open-ended questions because the former were easier to answer.

### The operation process

#### It is easy to forget at the end of the semester

*S7“We have so many teachers in one curriculum. We always forget their performance at the end of the term”.*

*E9“The time for evaluation should be advanced before exams so that the evaluation scale will not be affected by the difficulty of the tests”.*

Many students did not support the final evaluation. On the one hand, they usually forgot the teaching situation; on the other hand, they could not see any improvement in teaching. To avoid affecting their exams, the assessment time should be advanced. From the results of the questionnaire, 69.35% of the teachers held the opinion that each teacher’s content might be evaluated in a timely manner at the end of lessons, while 56.45 and 20.97% of them approved of conducting teaching evaluations at the end of term and at the mid-term, respectively. In addition, 6.45% of the teachers proposed having a continuous evaluation throughout the whole semester.

#### Incomplete information on the teaching evaluation system

*S2“Many teachers do not upload avatars or update them in a timely manner, and we are unable to match them one by one. Some experimental classes adopted group teaching. We did not take some of these classes, but we need to evaluate the teachers”.*

*S4“The mobile phone operation always flashes back. After evaluating a teacher, we must click save. Otherwise, all previous operation records will be invalidated”.*

Currently, SETs use online assessment at the end of the semester. According to our results, the students generally felt that this experience was poor and that the main obstacle was the lack of an ideal correspondence between the evaluation system and the real situation in class. The teaching evaluation system was limited to computer operation. The students looked forward to the development of mobile operating systems.

### The consequences of evaluation

#### Lagged feedback

*E7“Feedback is not provided in a timely manner, and only the final grades are given. I do not know what I did wrong”.*

*S10“As students, we have not received any feedback”.*

The feedback effect is unobvious or even deviated, affecting the continuous improvement in teaching quality. Based on our survey, the teachers hoped to obtain targeted results and expected that the evaluation results were protected and kept private. At the same time, the students hoped to obtain results that might be used as a basis for them to select courses and develop effective suggestions. In terms of comprehensive feedback, a majority of the teachers reported feedback but did not provide helpful guidance, accounting for 37.68% of our questionnaire survey sample. Additionally, 30.43% of the teachers thought that there was feedback and helpful guidance, while 27.54% of the teachers indicated that there was no feedback, and 4.35% of the teachers said that they were unclear. In other words, the effective feedback rate was still at a low level (Table 5).

**Table 5:** Teacher’s opinion about Feedback of teaching evaluation results(n, %)

Cases	Student evaluation	Supervisor\peer teacher evaluation	Comprehensive evaluation
No feedback	11(15.94%)	23(33.33%)	19(27.54%)
Have feedback\No guidance	30(43.48%)	16(23.19%)	26(37.68%)
Have feedback\Have guidance	25(36.23%)	25(36.23%)	21(30.43%)
Unclear	3(4.35%)	5(7.25%)	3(4.35%)
Total	69(100%)		

#### The rationality of the result application is questioned

*E11“I support the use of teaching evaluation results as the basis for selecting outstanding teachers, but they are not objective enough to use as a rigid index for professional promotion. The 50% selection rate is too low”.*

*E12“I suggest a decrease from 50% to 5% or 10%, mainly to discourage teachers who do not carefully prepare their lessons”.*

*E4“The faculty should monitor teachers whose SETs are less than 80 points to examine improvement in subsequent performance. What’s more, the degree to which teachers improve their teaching standards often depends on subjective initiative”.*

At present, evaluation results are applied in advanced selection, post appointment, momentary rewards, position promotion and other aspects related to the vital interests of teachers. The one-vote system (teachers with scores in the bottom 50% of the ranking have no chance of promotion) makes the evaluation results more sensitive for teachers. Nevertheless, a minority of teachers still support this policy, saying that it creates an atmosphere that values quality. For teachers with negative results, teacher training and monitoring are adopted to motivate them. According to our questionnaire survey, a majority of the teachers (95.65%) approved of applying evaluation results in selecting outstanding teachers, while others suggested using evaluation results in work assessment (*n* = 60, 86.96%), curriculum improvement (*n* = 57, 82.61%), professional promotion (*n* = 40, 57.97%), and rewards and punishment (*n* = 37, 53.62%).

## Discussion

Our study investigated the current issues in evaluations of teaching quality in undergraduate medical education from the perspective of multiple stakeholders. We extracted 29 open codes, 14 axial codes, and 4 selective codes through comprehensive analyses of qualitative and quantitative data, which are important elements in the evaluation process. The results of this research identified some relevant aspects of course evaluations reported in the literature [12, 16]. The main barriers include inadequate attention, unreasonable weighting, poor teaching facilities, an index without pertinence and appropriate descriptions, bad time-points, incomplete information on the system, lagged feedback, and disappointing result application.

Since the late twentieth century, universities have been ardently encouraging faculty research. In particular, in recent years, a growing number of universities have forced faculty members to increase their research output due to academic utilitarianization and commercialization [30]. Under such a policy orientation and financial incentives, teachers have made greater efforts with regard to their scientific research compared to their teaching, to say nothing of improving the quality of their teaching. In our interviews, many teachers said that they felt stressed in this atmosphere. Due to the weak culture of teaching quality, students tend to hold negative attitudes towards evaluations, relying on their “gut feeling” rather than using objective benchmarks of course quality [19]. As suggested by a survey conducted at Yanshan University, 52.6% of students evaluated teaching quality only to check their grades, only 27.8% thought that they always took evaluations seriously, 27.7% occasionally kept an active attitude, and another 5.6% never completed evaluations carefully [31]. Additionally, the lack of awareness of teaching process and curriculum structure for students may derail the effectiveness of evaluation. Students may give inflated ratings for all teachers, making it difficult to distinguish proficient teachers from less skilled teachers [32]. Moreover, students as subjects are currently assigned too much weight, resulting in further deviation of the results. Based on faculty evaluation practices, the University of Nebraska–Lincoln recommends that student evaluation scores should not be given undue weight since these scores can be easily manipulated and are slightly impractical [33]. A holistic framework that includes peer review, self-reflection and student feedback has been proven to be effective by the University of Oregon [34]. Our research results showed that it was appropriate to assign students as the subjects of evaluation a weight of approximately 50–60%.

The index system is a bridge between the subject and object of evaluation. If inappropriate indices are selected to gauge the quality of teaching, the evaluation results may drive inappropriate behaviours in universities [35]. Similar to Mao-hua Sun’s investigation, our study indicated that the existing evaluation system lacked pertinence to the discipline, the evaluation objective was insufficient, and the evaluation methods were simplistic [36]. Different subjects might have different definitions of high-quality teaching, and our study also implied the importance of selecting indicators that are in line with the subject’s cognitive level [37, 38]. It may be desirable to involve evaluators in the design of indicators rather than merely making their design the responsibility of teaching administrators. Different from previous studies, in our study, students showed a preference for closed-ended questions over open-ended questions, which may indicate that the subjective consciousness of students as evaluators needs to be further strengthened. Striking differences in teachers’, supervisors’ and students’ definitions of good teaching have been reported before [39]. However, the controversy among our participants over who should be responsible for student attendance really surprised us, which implies that it is necessary to redefine the roles and responsibilities of school staff. To improve the pertinence of the index system, some universities divide the indices into compulsory and optional, allowing teachers to choose based on the real situation [40], while other universities develop different indicators for different courses [41].

At the same time, defective teaching equipment exerts a great influence on evaluation results. However, this point has rarely been mentioned in previous studies. One interviewee was quoted as saying, “when device crashes, it not only causes a waste of valuable class time but also seriously affects student satisfaction and teaching quality”. According to a study by Nanchang University, 58.82% of staff stated that they often encountered equipment breakdowns; for instance, a U disk was not recognized, the network was not connected, and the screen was black [42]. Such instances may be because insufficient funds are invested and the maintenance of infrastructure is inadequate [43]. Additionally, the students surveyed complained about the defects of the evaluation system. If we can provide complete and consistent information on the system and smooth the operation process, the student participation rate may increase [44, 45]. These measures would also be beneficial for solving the problem posed by the fact that for students, it is “easy to forget at the end of the semester” because there would be enough information on the system to refer to. The World Federation for Medical Education (WFME) regards education information as an important resource for improving teaching quality [46]. However, to perfect teaching equipment in the future, a long period of time is still necessary.

The plan-do-check-act (PDCA) cycle is a tool for promoting quality improvement. According to PDCA theory, feedback is an important link for realizing the closed-loop management of teaching evaluation and continuous quality improvement. Based on our results, the feedback problems mainly concentrated on time, content and methods. Many teachers said that the feedback was too slow to adjust their behaviours in a timely manner. Moreover, 30% of the teachers reported that they received no feedback whatsoever. On the other hand, the overly simplistic content of feedback also confused the teachers. It is often the case that only a total evaluation score is given, and aspects for which there is good performance or poor performance cannot be judged. The seriousness of this problem has also been highlighted in other studies [47, 48]. Despite agreement on the value of evaluations, differences between teachers’ and students’ perceptions emerged in terms of confidentiality and whether the results should be made public. A few teachers thought that their results should be kept private, while the students believed that it was their right to know the results. Some studies have shown that for average teachers, it is better to use confidential methods. For teachers with higher scores, their inspiring results should be open to the whole school to set a typical example and motivate underperforming teachers to improve [49]. To reach a consensus between teachers and students, more research into the effectiveness and fairness of feedback is warranted. To some degree, how the individual perceives the consequences of evaluation may be more important than the outcome itself [50]. Regarding result application, most teachers preferred incentives over negative consequences. Compared with external incentives, the effect exerted by recognition from within the teaching profession itself and the pursuit of quality improvement is stable and persistent. However, once teachers are unfairly treated in salary or promotion, their enthusiasm for teaching evaluations may be frustrated, affecting their recognition from within the profession. This phenomenon implies that evaluation results should be used for positive encouragement, not as a punitive measure.

**Fig. 1:**
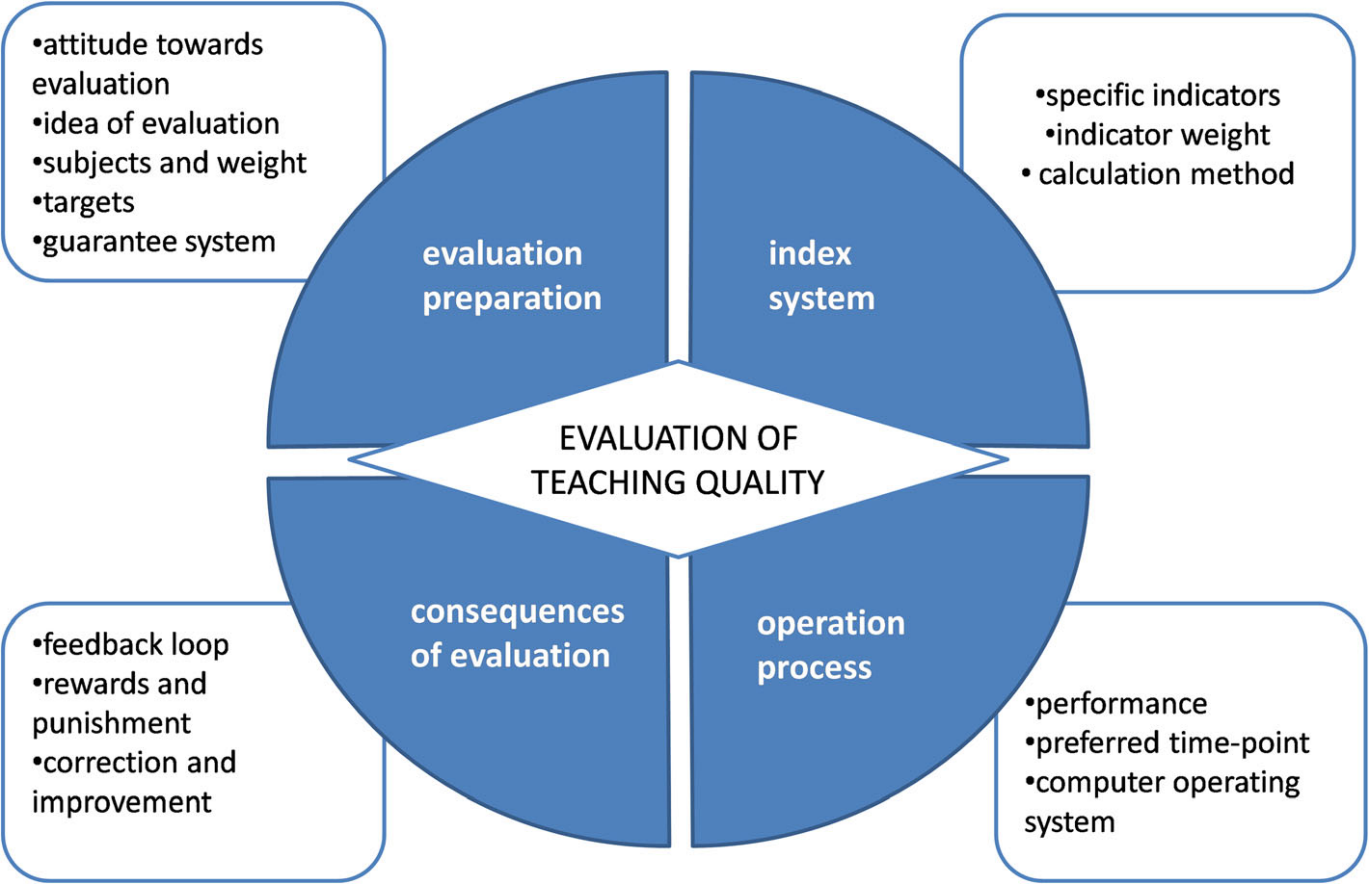
Model of barriers to obtaining reliable results from evaluations of teaching quality

The main value of this study lies in its two contributions. To the best of our knowledge, this research is the first comprehensive qualitative and quantitative study to reflect the current problems in evaluations of teaching in undergraduate medical education. According to our investigation, there are 4 obstacles that may hinder the successful implementation of evaluations: evaluation preparation, the index system, the operation process and the consequences of evaluation. Some of our findings are consistent with commonly accepted concerns in the teaching evaluation process. Additionally, data from the perspective of multiple stakeholders add several fresh opinions to the literature on this topic. Evaluating teaching quality involves different subjects. All subjects have their own interest considerations and value appeals, and with respect to their feedback, they affect each other through sophisticated behaviours. The results of this study can be used as a reference to design a teaching quality evaluation framework and system, which may arouse the interest of managers or leaders who need to be aware of the assumptions and confounders underlying the evaluation scores in an institution similar to ours. In general, adequate evaluation preparation and a scientific index system are prerequisites for obtaining objective and fair results. Meanwhile, a reasonable and convenient operation process guarantees the smooth implementation of evaluations, while precise result processing and timely feedback further ensure the significance of evaluation work, resulting in a virtuous circle (Fig. 1).

However, some limitations of this study should also be noted. First, the 16 experts, 69 teachers and 12 students who were involved in this study form a limited sample pool. Collecting information from a variety of stakeholders will improve our understanding of evaluations of teaching quality. Second, although many obstacles in the teaching evaluation process were identified, the relationships between these obstacles remain unclear. How to solve these existing problems should definitely be studied in further research.

## Conclusions

This study reveals the barriers to obtaining objective and accurate results during the process of evaluating teaching quality in undergraduate medical education. In doing so, the opinions of different stakeholders on this topic were collected using mixed methods. It should be noted that this study is conducted in the context of China. Hopefully, the findings of this study can improve our understanding of evaluators’ attitudes and evaluation process. Educators need to be aware of various issues that potentially impact the final results when designing the evaluation system and interpreting the evaluation results. More research on solutions to these problems and the development of a reasonable evaluation system is warranted.

## Supplementary information

**Additional file 1.** Questionnaire for evaluation of teaching quality in medical education.

## Data Availability

The datasets used and/or analyzed during the current study are available from the corresponding author on reasonable request.

## Abbreviations

SETs: Student evaluations of teaching
WFME: World Federation for Medical Education
PDCA: Plan-do-check-action cycle
