# Supplementary material for: Barriers to obtaining reliable results from evaluations of teaching quality in undergraduate medical education
Source: BMC Med Educ. 2020 Sep 29;20:333. doi: 10.1186/s12909-020-02227-w (PMC7523339; doi:10.1186/s12909-020-02227-w)
Supplement: Supplementary file 1 — Additional file 1. Questionnaire for evaluation of teaching quality in medical education. [file 12909_2020_2227_MOESM1_ESM.docx]

Additional File

**QUESTIONNAIRE FOR EVALUATION OF TEACHING QUALITY IN MEDICAL EDUCATION**

Thank you for agreeing to complete this questionnaire.

This questionnaire mainly investigates your views on the current teaching evaluation in medical education. The survey will take approximately 2 minutes to complete.

The data in this form is collected in a way that does not allow individuals to be identified. All your answers will be treated as confidential and will only be used by the research team.

**Personal details**

1. Your gender ( ) A. Male B. Female

2. Your job title ( ) A. lecturer B. Associate Professor C. Professor

**Views on teaching evaluation**

3. In your opinion, which of the following subjects should be included in the evaluation of teaching quality? ( )

You can choose more than one answer.

A. Students B. Teacher himself/herself C. Peer teachers D. Supervisors

C. Leaders D. Teaching management staff E. Others

4. How do you think the weight of the evaluation subjects selected in the preceding question are allocated?

For the unselected options in the previous question, fill in 0, and the total weight of all options is 100% .

Fill in the blank%.

A. Students_____ B. Teacher himself/herself____

C. Peer teachers____ D. Supervisors____

E. Leaders____ F. Teaching management staff____ G. Others____

5. In which ways do you think the results of evaluation should be applied? ( )

You can choose more than one answer.

A. Work assessment B. Rewards and punishments C. Title promotion

D. Excellent selection E. Curriculum improvement F. Others

6. When do you think is the appropriate time for teaching evaluation? ( )

You can choose more than one answer.

A. Midterm B. End of term C. End of the lecture

D. Whole semester E. Others

7. What do you think of the current evaluation index system?

Choose the answer that you think is the most appropriate

A. Very good B. Good C. Just so-so D. Poor E. Very poor

8. Do you think the number of students has an impact on the results of teaching evaluation? ( )

Choose the answer that you think is the most appropriate.

A. The more the number of students, the higher the score

B. The more the number of students, the lower the score

C. Almost no effect

9. Do you get feedback on the results of student evaluation? ( )

Choose the answer that you think is the most appropriate

A. No feedback B. With feedback, no guidance

C. With feedback, with guidance D. Others

10. Do you get feedback on the results of supervisors or peer teachers evaluation? ( )

Choose the answer that you think is the most appropriate

A. No feedback B. With feedback, no guidance

C. With feedback, with guidance D. Others

11. Do you get feedback on the comprehensive evaluation results? ( )

Choose the answer that you think is the most appropriate

A. No feedback B. With feedback, no guidance

C. With feedback, with guidance D. Others

12. How do you feel about the current teaching evaluation work? ( )

Choose the answer that you think is the most appropriate

A. Very good B. Good C. Just so-so D. Poor E. Very poor

13. Please write here any other comments or suggestions about teaching evaluation in medical education that you would like to make:

_________________________________________________________________________________________

THANK YOU FOR YOUR PARTICIPATION!
